# Using social media influencers to increase knowledge and positive attitudes toward the flu vaccine

**DOI:** 10.1371/journal.pone.0240828

**Published:** 2020-10-16

**Authors:** Erika Bonnevie, Sarah D. Rosenberg, Caitlin Kummeth, Jaclyn Goldbarg, Ellen Wartella, Joe Smyser

**Affiliations:** 1 Department of Health Communications, The Public Good Projects, New York, NY, United States of America; 2 Marketing Department, The Public Good Projects, New York, NY, United States of America; 3 Northwestern University School of Communications, Evanston, IL, United States of America; Newcastle University, UNITED KINGDOM

## Abstract

Seasonal influenza affects millions of people across the United States each year. African Americans and Hispanics have significantly lower vaccination rates, and large-scale campaigns have had difficulty increasing vaccination among these two groups. This study assessed the feasibility of delivering a flu vaccination promotion campaign using influencers, and examined shifts in social norms regarding flu vaccine acceptability after a social media micro influencer campaign. Influencers were asked to choose from vetted messages and create their own original content promoting flu vaccination, which was posted to their social media pages. Content was intentionally unbranded to ensure that it aligned with the look and feel of their pages. Cross-sectional pre- and post-campaign surveys were conducted within regions that received the campaign and control regions to examine potential campaign impact. Digital metrics assessed campaign exposure. Overall, 117 influencers generated 69,495 engagements. Results from the region that received the campaign showed significant increases in positive beliefs about the flu vaccine, and significant decreases in negative community attitudes toward the vaccine. This study suggests that flu campaigns using a ground-up rather than top-down approach can feasibly reach at-risk groups with lower vaccination rates, and shows the potentials of using an influencer-based model to communicate information about flu vaccination on a large scale.

## Introduction

Every year, seasonal influenza affects millions of people in the United States. During the 2018–2019 influenza (or flu) season, the flu was involved in up to 20 million hospitalizations and up to 61,000 deaths in the United States [1, 2]. One of the most effective ways of preventing the flu is by getting a flu vaccine every year and the U.S. Centers for Disease Control and Prevention recommends all individuals 6 months of age and older receive the flu vaccine each year [1]. Yet despite the seriousness of the flu, many people do not get vaccinated.

Studies have shown racial and ethnic disparities in flu vaccination rates. Despite the implementation of large-scale flu vaccination campaigns across the U.S., vaccination coverage among African Americans and Hispanics has remained low, with recent flu vaccination coverage for African Americans at 32.3% and for Hispanics at 28.4%, compared to over 40% vaccination coverage for their White and Asian counterparts [3–5]. Negative perceptions toward the flu vaccine may at least partially explain the low flu vaccination coverage, which may lead to social norms that discourage vaccination [6, 7]. Studies have shown that African Americans are more likely than other groups to have higher risk perceptions of the flu vaccine and its potential side effects, to mistrust the medical community and government involvement in ensuring safety of the vaccine, and to be uninformed regarding the benefits of flu vaccination [8–10]. Hispanics are less likely than other groups to believe that the flu vaccine is effective, and more likely to fear vaccine side effects [11, 12]. Both African Americans and Hispanics are more likely than other groups to believe that the flu vaccine is simply a way for physicians to make a profit [13]. Conversely, individuals who report that a majority of people around them receive the flu vaccine are in turn more likely to intend on becoming vaccinated themselves [14]. All of these factors potentially lead to differential vaccination coverage among races and ethnicities within the U.S., and ultimately, a higher morbidity and mortality for African Americans and Hispanics from flu and flu-related illness [7, 15].

Given these disparities, there is a need for more information on strategies to reach these populations with positive messages about flu vaccination, using methods that can be applied on a large-scale. Researchers have suggested some technology-based strategies to increase vaccination rates, including digital surveillance technology via electronic medical records, or communications through text messages, email and social media [16–19]. However, many of these methods have not been tested on a large scale for flu vaccination, have not shown effectiveness (particularly among the populations identified above), and may depend on individuals meeting the researchers where they are—for example, on an app [19]. While researchers have examined the relationship between the use of social media and flu vaccination, there is a lack of information on using social media influencers to deliver health messaging about flu vaccination [20, 21]. This study adds new evidence to show the potential uses of a social media influencer model. Social media influencers are individuals on social media who have built a credible reputation and following, oftentimes in a specific niche topic area [22]. For over a decade, the marketing sector has used social media influencers as a cost efficient and effective way to sell products [23]. Social media influencers are just starting to be used in public health, with research beginning to show their promise in promoting various health behaviors; social media influencers have shown promise in achieving high levels of digital engagement and positive health outcomes, with researchers calling for more investigation into applications of this model for other behaviors [24–27]. The present study applies these theories to a campaign that promotes positive views of the flu vaccine.

From October 2018 to March 2019, The Public Good Projects (PGP) implemented a digital campaign using social media micro influencers to increase knowledge and positive attitudes toward the flu vaccine among African Americans and Hispanics living in Kaiser Permanente service regions. The campaign employed user-generated content from social media micro influencers whose followers disproportionately represent the campaign’s target audiences in the areas that received the campaign. For the purposes of this campaign, a micro influencer was considered someone with 500 to 10,000 followers on at least one social media account. Micro influencers were utilized because these individuals may be more likely to be perceived as friends or aspirational peers than celebrities or influencers with significantly larger followings. Friends and peers may be well positioned to impact perceptions of vaccines at the interpersonal level, within models of behavior change such as the Social Ecological Model [28]. Micro influencers often have trusting relationships with their followers, who often reside in a specific geographic region, and these influencers are unlikely to have large, multi-state followings [29]. The goals of the campaign were to use influencers to deliver messages about the flu vaccine as a way of shifting social norms toward embracing a positive view of the flu vaccine. To date, this is the largest influencer-driven flu vaccination campaign focused on reaching African American and Hispanic communities in the U.S. The objectives of this study were to assess the feasibility and potential acceptability of delivering a flu vaccination promotion campaign through the use of influencers, and to describe any potential differences in flu-related attitudes before and after implementation of the social media influencer campaign.

## Methods

Influencers were recruited through influencer recruitment software, which contained information on each individual’s geographic reach and proportion of followers who were African American or Hispanic. To ensure that influencers had legitimate followers and were not engaging in “influencer fraud” (for example by purchasing followers), influencer software provided a credibility score which estimated how many followers were actively engaged as well as a follower growth chart to detect spikes in followings that may reflect fraudulent behaviors. These measures allowed PGP to be confident that all influencers who took part in the campaign were not engaging in fraud to grow followers. The platform provides the ability to filter influencers by those who fit demographic and geographic targeting criteria, from their list of over 2 million influencers who are signed up for the platform. An open call was sent to all influencers who met the eligibility criteria, including that their following consisted of a primarily African American or Hispanic audience, located within the area that received the campaign. It is therefore not possible to determine the percentage who viewed the open call to participate in the campaign and refused to take part, or the reasons for non-participation.

Influencers selected for this study created personal messages, images, and/or videos promoting the flu vaccine and posted them on their social media accounts. Influencers were asked to choose from a selection of previously-vetted messages pertaining to flu vaccination and create their own original, user-generated content, in either English or Spanish, promoting flu vaccination using one of those message prompts (for example, “Need a good reason to get a flu shot? How about to protect not just yourself but those you care about most? I’m getting the flu shot this year for my daughter. Check out stopflu.org to find out where to get a flu shot near you. #stopflu”). Due to negative connotations associated with the word “vaccination,” all posts referenced the “flu shot.” Message prompts were changed on a monthly basis and focused on dispelling common myths about the flu vaccination as well as general encouragement to initiate flu vaccination.

Influencers were asked to ensure that their post referenced at least one vetted fact that was provided to them. Facts were pre-selected and related to the following eight categories, which corresponded to common gaps and misconceptions about the flu vaccine and which formed the basis for this study’s evaluation questions: 1. The importance of protecting yourself, your family, and the community; 2. Everyone needs a flu shot, even those who are healthy; 3. Addressing myths (i.e., ‘the flu shot cannot give you the flu’); 4. Highlighting convenience of flu shot locations; 5. The seriousness of the flu; 6. It’s “never too late” to get the flu shot; 7. Safety and efficacy of the flu shot; and 8. Minimal side effects of the flu shot, especially compared to being infected with the flu. While the eight prompt categories were used throughout the season, specific messages changed as the flu season progressed. From September to November, posts focused on preparation; from December to January, posts focused on getting the flu vaccine during flu season; and from February to March, posts focused on the importance of getting the flu vaccine even late in the season. To ensure that prompts were relevant and engaging, influencers were asked to create posts that tied into relatable moments and holidays.

Influencers were not required to respond to comments on their posts, and all responses to engagements with the posts were at the discretion of the individual influencer. Influencers posted one message across each of the platforms on which they were active as an influencer (Facebook, Instagram, and/or Twitter). Each message included a link to the campaign website, stopflu.org. The website complemented influencer posts and inspired action by providing information about where flu vaccination was offered. Upon reaching the website, a user was given two paths: clicking either “I’m a Kaiser Permanente member” or “I’m a member of another system.” Kaiser Permanente members were sent to the health system’s “Health & Wellness” page, where they entered their region to locate a flu clinic. Non-Kaiser Permanente members were sent to a vaccine finder page, where they were able to locate their own local clinic.

Compensation for influencers depended on reach and influence; those with a larger reach and more influence received more compensation. Influencers were compensated up to $360 for their participation, with an average of $84.46 per influencer. The campaign spent approximately $15,000 on payments to influencers. Before participating, influencers were required to pass a 3-month retrospective review of all public social media posts. Vetting criteria included no promotion of alcohol, tobacco, firearm products, or inflammatory or offensive posts of a sexual, political, or bigoted nature.

Content produced by influencers was intentionally unbranded, a term referred to as “native advertising” [30]. Using unbranded content and relying on the effectiveness of tailored messaging is an established practice in marketing, but is relatively untested within the public health sphere [31, 32]. This method of advertising promotes ideas related to a specific behavior change in order to have an effect on behavior that is beneficial to the brand (or in this case, the health behavior). Native advertising matches the message being promoted with the style of content already on the page or individual’s social feed where the promoted message will appear. Native advertising is designed to motivate individuals to adopt a certain behavior, without relying on awareness of campaign brand names. Individuals are more likely to spend time viewing native content compared to content that are clearly advertisements [33, 34].

To describe potential differences in flu-related attitudes before and after the campaign, two cross-sectional online evaluation surveys were conducted that included purposively-recruited respondents across campaign and control areas. Campaign areas include eight regions in which the campaign was delivered (corresponding to the regions in which Kaiser Permanente has an active presence): Northern California, Southern California, Colorado, Georgia, Hawaii, Mid-Atlantic States (Maryland, Virginia, and Washington D.C.), Oregon, and Washington. Control areas include the following eight states, chosen to resemble campaign region demographics as closely as possible: Alabama, Arizona, Nevada, New Mexico, New York, Oklahoma, Pennsylvania and South Carolina. The baseline survey was conducted prior to the U.S. flu season, from August 22, 2018 to September 21, 2018. The follow-up survey was conducted after the end of the typical flu season, from March 1, 2019 to April 8, 2019. All respondents included in this study were recruited to participate via research panels from Qualtrics, a research panel company, and were selected to match their representative populations as much as feasibly possible given the recruitment method. Eligibility criteria included being 18 to 64 years of age, Hispanic and/or African American, English or Spanish-speaking, and currently living within one of the identified campaign or control areas. Race and ethnicity were presented as two separate questions, and respondents could choose all races that applied to them. After reading a consent form explaining the nature of participation, each individual was asked to provide electronic informed consent to continue with the survey, which confirmed their enrollment. This study was conducted in accordance with the Code of Ethics of the World Medical Association (Declaration of Helsinki) for experiments involving humans and was reviewed by IntegReview and determined to be exempt from IRB review.

Evaluation survey questions were derived guided by the Theory of Planned Behavior, which states that behavior is determined by attitudes, perceptions of social norms, and beliefs of control or self-efficacy to accomplish the intended behavior change [35]. The survey assessed respondent demographics and examined social norms regarding the flu vaccine, both before and after the campaign. The survey instrument utilized existing standardized and validated measures of knowledge, attitudes, and reported and intended behavior related to the flu vaccine derived from flu-related surveys conducted across the U.S. (such as the National H1N1 Flu Survey and surveys from the National Foundation for Infectious Diseases), as well as academic research from noted researchers and organizations in the field [36–39]. To determine exposure to the campaign, at follow-up, respondents were asked if they had seen posts that promoted the flu vaccine on their social media pages. To evaluate the feasibility of using an influencer-based campaign to promote flu vaccination, an examination of digital metrics was undertaken to determine the level of engagement generated by influencer posts. Results from the questions in the online survey were compared for various groups (i.e., baseline versus follow-up; exposure to posts versus no exposure to posts) using a 2-sided Pearson Chi-squared test (alpha was set at 0.05), and data were analyzed using IBM SPSS Statistics. An a priori power analysis was conducted to ensure sufficient sample size for statistical comparisons. Google Analytics was used to examine campaign digital metrics. Potential campaign reach was calculated by adding the total number of followers of all influencers.

## Results

### Feasibility of the campaign

The 117 recruited influencers resided in the following locations: Colorado (n = 10), Georgia (n = 11), Hawaii (n = 10), the Mid-Atlantic States (10 in Maryland, 10 in Washington D.C. and 10 in Virginia), Northern California (n = 13), Oregon (n = 11), Southern California (n = 21), and Washington State (n = 11). Influencers involved in the campaign showed a variety of topic interests typically displayed on their page, with the largest group (31%) being influencers who typically post about parenting, followed by travel influencers (10%). Others included fashion, photography, or health and wellness influencers (all comprising less than 10% of influencer interests). A majority of influencers were female (77.7%), compared to males (19.4%) and couples whose accounts featured multiple people (2.9%). Across all states, 59.4% of influencers reached predominantly African American followers, compared to 36.8% of influencers who reached predominantly Hispanic followers, and 3.8% who reached both.

Throughout the campaign period, the influencers reached a potential of 9.9 million individuals on social media, and generated 69,495 engagements (likes, shares, or comments). Approximately 86% of influencers chose to post an image, 19% chose to post text, and 5% chose to create a video. See S1 File for examples of influencer posts. English posts reached a potential of 8.4 million social media users and generated 49,471 engagements, while Spanish posts reached a potential of 1.5 million individuals and generated 20,424 engagements. The results of the campaign demonstrated that influencer posts generated high levels of engagement, on par with general marketing industry standards. In terms of the engagement rates, Spanish-language posts appeared to be more engaging, with 20,000 engagements across a potential reach of 1.5 million, compared to 50,000 engagements across a potential reach of 8.4 million for English-language posts.

The website attracted 16,064 unique homepage views and page views on the Vaccine Finder page; 76.0% of those who visited the Vaccine Finder page used the tool and entered their zip code to find their nearest vaccination site. A total of 809 unique zip codes were entered into the Vaccine Finder. Of those, 770 were from a state exposed to the campaign, 23 were from states not exposed to the campaign and 16 were invalid. The average time spent on the Vaccine Finder page was 3 minutes and 13 seconds.

### Evaluation survey demographics

A total of 4,904 respondents completed the baseline survey (Table 1). Of those, 2,435 resided in campaign areas and 2,469 resided in control areas. The follow-up survey was conducted among 5,447 respondents, with 2,719 residing in campaign areas and 2,728 residing in control areas. More than 50% of respondents in both the baseline and follow-up surveys were in the age range of 18 to 35. Gender distribution was similar between the two survey periods, with slightly more female respondents across both time periods and regions. At both baseline and follow-up, there were more Hispanic respondents in the campaign areas compared to the control areas. Conversely, there were more African American respondents in the control areas compared to campaign areas.

**Table 1:** Demographics at baseline and follow-up, campaign area vs. control ^a^.

		Campaign Area		Control	
		Baseline (n = 2,435)	Follow-Up (n = 2,719)	Baseline (n = 2,469)	Follow-Up (n = 2,728)
Age Group					
	18–25	34.2% (833)	27.1% (738)	31.7% (782)	27.7% (756)
	26–35	31.4% (764)	28.2% (768)	31.1% (768)	27.6% (753)
	36–45	18.7% (455)	19.5% (529)	19.5% (482)	18.9% (515)
	46+	15.7% (383)	25.2% (684)	17.7% (437)	25.8% (704)
Gender					
	Male	46.3% (1128)	44.2% (1201)	48.2% (1191)	46.7% (1274)
	Female	52.6% (1282)	54.5% (1481)	51.1% (1261)	52.5% (1432)
	Other	0.6% (15)	1.0% (27)	0.3% (7)	0.3% (7)
	Prefer to not say	0.4% (10)	0.4% (10)	0.4% (10)	0.5% (15)
Hispanic		66.4% (1618)	61.5% (1673)	56.8% (1403)	53.8% (1468)
Race ^b^					
	White	40.5% (985)	36.9% (1002)	36.7% (906)	28.7% (784)
	Black	38.9% (947)	43.8% (1192)	49.1% (1213)	53.8% (1469)
	Asian	3.2% (78)	3.3% (90)	1.5% (37)	1.6% (43)
	Native American/ Alaska Native	5.0% (121)	4.8% (131)	3.5% (86)	3.6% (99)
	Hawaiian/ Pacific Islander	3.0% (74)	1.6% (43)	1.1% (26)	0.8% (22)
	Other Race	12.2% (297)	12.9% (350)	9.7% (239)	12.6% (344)
	Prefer to not say	5.5% (134)	4.3% (117)	4.0% (99)	4.1% (112)

^a^ Data are %(n).

^b^ Race variable are not mutually exclusive. Total N and (%) may not add up to the stratum specific sample sizes.

### Previous flu and vaccine history

More than 50% of respondents in the campaign group reported that they receive the flu vaccine every year or most years, while 20% reported they receive the vaccine during some years, and greater than 25% reported that they never get the flu vaccine. These figures were not significantly different between baseline and follow-up for either the campaign or control groups (Table 2). Previous flu vaccination coverage was similar, but differed significantly, between the campaign and control areas (Table 2). At baseline, the campaign and control groups were not significantly divergent in flu vaccine behaviors and intentions at baseline. At follow-up, respondents were asked if they got the flu vaccine during the previous flu season. Respondents in the campaign region reported slightly higher vaccination rates at follow-up, with nearly 45% receiving the vaccine in the campaign group and 42% receiving it in the control group. This difference was not significant.

**Table 2:** Previous flu and vaccine history at baseline and follow-up, campaign area versus control^a^.

		Campaign Area			Control		
		Baseline	Follow-Up	*p*	Baseline	Follow-Up	*p*
How often do you get the flu vaccine?				0.626			0.591
	Every Year or Most Years	55.6% (1353)	54.4% (1478)		51.0% (1258)	51.0% (1392)	
	Some Years	19.3% (470)	20.2% (550)		20.3% (500)	19.2% (525)	
	Never	25.1% (612)	25.4% (691)		28.8% (711)	29.7% (811)	
Are you planning on getting the flu vaccine for the upcoming flu season (fall and winter 2018–2019)? ^b^							
	Yes	34.3% (835)			33.5% (826)		
	No	26.1% (636)			28.5% (703)		
	Don't know	16.2% (394)			15.2% (376)		
	Already been vaccinated	23.4% (570)			22.8% (564)		
In the past 6 months, did you get the flu vaccine? ^c^			44.4% (1206)			42.0% (1146)	

^a^ Data are % (N).

^b^ Question presented during baseline survey only.

^c^ Question presented during follow-up survey only.

### Social norms regarding the flu and flu vaccine

In the campaign area, several measurements of specific knowledge and positive attitudes toward the flu vaccine were statistically significantly higher at follow-up than at baseline (Table 3). In particular, the campaign area had significantly higher percentages at follow-up versus baseline of those who: believe it is never too late to get a flu vaccine (p < .05), disagreed that healthy people do not need to get the flu vaccine (p < .05), believe the government closely monitors the safety of the flu vaccine (p < .05), and agreed that respondents would get the flu vaccine if everyone else was getting it (p < .05). Across these measures, the control area did not show a significantly higher percentages of agreement from baseline to follow-up. The campaign group showed a significantly lower percentage of those who believe that the side effects of the flu vaccine are worse than the flu (p < .05) from baseline (34%) to follow-up (32%), while the control group showed a higher percentage for this measure (p < .05) from baseline (35%) to follow-up (37%).

**Table 3:** Statements of agreement for social norms regarding the flu vaccine at baseline and follow-up, campaign area versus control^a^.

	Campaign Area			Control		
	Baseline	Follow-Up	*p*	Baseline	Follow-Up	*p*
It’s never too late in the flu season to get the flu vaccine.	59.8% (1456)	63.0% (1713)	*0*.*010*	59.6% (1472)	61.3% (1671)	*0*.*484*
Healthy people don’t need to get the flu vaccine. ^b^	57.0% (1389)	61.0% (1659)	*0*.*012*	56.7% (1401)	58.4% (1593)	*0*.*172*
I would get the flu vaccine if everyone else was getting it.	34.6% (842)	37.2% (1012)	*0*.*040*	34.9% (861)	34.9% (952)	*0*.*02*
The side effects of the flu vaccine are worse than the flu.	33.8% (823)	31.8% (864)	*0*.*019*	35.4% (874)	36.5% (995)	*0*.*037*
The government closely monitors the safety of the flu vaccine.	43.1% (1049)	46.1% (1254)	*0*.*048*	43.1 (1064)	44.8% (1221)	*0*.*456*
My friends think the flu vaccine is not effective.	33.2% (808)	30.1% (818)	*0*.*016*	32.1% (793)	32.0% (874)	*0*.*095*
My family thinks the flu vaccine is not safe.	30.7% (747)	30.0% (817)	*0*.*029*	33.5% (826)	33.4% (912)	*0*.*018*
My friends think the flu vaccine is not safe.	30.1% (733)	29.1% (790)	*0*.*037*	30.9% (762)	30.2% (825)	*0*.*234*
My family thinks they’re not at risk of getting the flu.	31.0% (754)	29.6% (805)	*0*.*034*	31.1% (769)	29.9% (815)	*0*.*050*

^a^ Data are % (n).

^b^ Data represents respondents who reported disagreement; all other measures represent respondents who reported agreement.

To understand perceptions of community-wide social norms, questions were also asked to gauge perspectives on family and friends’ attitudes toward the flu vaccine. Within the campaign area, there were several statistically significant decreases at follow-up versus baseline which were not replicated in the control area, including for questions around friends thinking the flu vaccine is not effective and friends thinking the flu vaccine is not safe (both p < .05).

### Differences among respondents exposed to posts

Table 4 displays an examination of differences between those who reported that they had seen a message promoting the flu vaccine on social media and those who did not within the campaign region (Table 4). At follow-up, 14.1% of respondents in the campaign region reported seeing positive flu promotion posts from someone they follow on their social media accounts. Those who had seen a flu promotion post on social media reported significant differences across various measures, including higher reported vaccination coverage compared to those who had not seen flu promotions (50.9% vs 43.3%), and significantly more often agreed that the vaccine is effective (58.2% vs 46.8%), that the vaccine is the best way to protect others from the flu, (67.1% vs 55.5%), and that the vaccine is worth the time and effort (67.1% vs 59.1%).

**Table 4:** Sub-analysis of exposure to flu promotion posts on social media at follow-up^a^.

	Exposure to Posts	No Exposure to Posts	*p*
I received the flu vaccine in the past 6 months.	50.9% (195)	43.3% (1011)	.*005*
The flu vaccine is safe for most people.	71.3% (273)	67.8% (1584)	.*176*
The flu vaccine is effective.	58.2% (223)	46.8% (1093)	*<* .*001*
It’s never too late in the flu season to get the flu vaccine.	67.1% (257)	62.3% (1456)	.*073*
Getting the flu vaccine is the best way to protect myself against the flu.	61.9% (237)	56.7% (1324)	.*056*
Getting the flu vaccine is the best way to protect others against the flu.	64.2% (246)	55.5% (1296)	.*001*
I would get the flu vaccine if everyone else was getting it.	48.0% (184)	35.4% (828)	*<* .*001*
Getting the flu vaccine is worth the time and effort.	67.1% (257)	59.1% (1381)	.*003*

^a^ Data are % (N). Table includes follow-up data for respondents who reported exposure to flu vaccination promotion posts in the campaign region only.

## Discussion

Results from this study of a campaign to affect social norms regarding seasonal flu vaccine among African Americans and Hispanics demonstrated a greater improvement in knowledge and positive perceptions of the flu vaccine among respondents sampled from the campaign area versus those in the control area in the post- campaign follow-up survey. Notably, respondents in the campaign area reported significantly higher agreement with social norms and perceptions of community attitudes conducive to receiving the flu vaccine than those in the control area at follow-up. Additionally, the differences detected in the campaign group were generally significantly higher in the follow-up period versus baseline. Finally, at follow-up, those in the campaign area who reported exposure to campaign posts were significantly more likely to have received the flu vaccine and report positive flu vaccine perceptions than those who did not report exposure to campaign posts.

Regarding the feasibility of using an influencer-based campaign to promote flu vaccination, the examination of digital metrics demonstrated high levels of engagement, signaling that this type of campaign can reach large numbers of people with a relatively limited amount of resources. Although it was not a not a main focal point of this study, another study undertook a qualitative analysis of comments on Stop Flu influencer posts over the course of two years of implementation, finding that on average 94% of comments that were made on influencer posts were of a positive nature [40]. This finding suggests that individuals will engage in a positive way with vaccine promotion messaging if it is presented from individuals that they already admire or follow. This is particularly important because flu vaccination (and vaccination in general) is a topic that is often subject to heavy debate, and digital campaigns can easily become grounds for the spread of false information and negative sentiment from individuals opposed to vaccinations [41–43]. The health community must react to these negative trends by utilizing new technology and innovative methods to communicate information in places that are less likely to be flooded with anti-vaccination messages, and in ways that will most resonate with at-risk audiences. Targeted messaging is also a critical piece of native advertising and producing effective behavior change campaigns. This study shows that targeted messaging for flu vaccination can be engaging for both English and Spanish–speaking populations. We theorize that the higher rate of engagement within the Spanish-speaking community may result from individuals not being accustomed to seeing health information in Spanish and in a style that resonates with them, so they felt more compelled to engage with the content, compared to those who viewed the English-language content.

In addition to showing the potential to reach African Americans and Hispanics in both English and Spanish, this study revealed other potential audiences that may be ideal to target for future flu vaccination campaigns. Baseline results from the campaign area showed that nearly 25% of respondents had already received the flu vaccine for the upcoming year, while just over 30% intended to do so, and around 15% were unsure. However, the follow-up showed final vaccination coverage around 45% of the sample. This suggests there are at least two groups that may be an ideal audience to target for future campaigns: those who intend on getting the vaccine (but do not), and those who are unsure if they will get the vaccine. Future studies should examine the feasibility of using an influencer-driven model to reach that group who express intention or hesitancy, but may be more receptive to receiving the vaccine. This methodology also has promising implications in communicating information about other topics that are hotly debated, including other non-seasonal vaccinations, particularly in light of recent measles outbreaks [44].

There are some limitations stemming from employing a cross-sectional panel-based survey. Since the panel company cycles panel participants, it is not possible to conduct a longitudinal study to determine whether a specific group of individuals were affected directly by the campaign. Therefore, all results are meant to demonstrate and test differences among samples at two unique timepoints. This was accomplished by selecting pre- and post- campaign samples of the population that were as similar to each other as possible on major characteristics and also as similar to their represented populations as was possible. While we acknowledge that these samples were not fully representative of the population or perfectly comparable to each other (the baseline and follow-up samples differed by age and race, but not on age), through triangulation of data, we believe our campaign did have the intended effect on the larger population, as analyses revealed that those who reported exposure to a campaign were more likely to report positive flu vaccination perceptions and, indeed, to receive a flu vaccine than those who were not exposed to a campaign. However, we were not able to control for all confounding factors, such as the level of participant vaccine education or the presence of vaccine drives in campaign or control communities. Survey respondents may show desirability bias or may not represent the general audience. This limitation may have been mitigated by the fact that panel participants are routinely cycled in and out of panels to avoid creating a pool of professional survey takers. The fact that the survey was completed online may have made respondents more likely to provide their honest opinions.

Other limitations of this study include that the severity of the flu season can affect changes in the constructs measured in the survey. However, data on the percentage of visits to outpatient clinics for Influenza-Like Illness (ILI) and the percentage of deaths resulting in pneumonia or influenza (measurements used to determine severity) presented similar results for both the campaign and control region, suggesting that both regions experienced similarly severe flu seasons [45, 46]. Although influencers were chosen based on their number of followers within campaign areas, it is possible that social media users outside the campaign areas also viewed their content. This result would be considered non-problematic in non-control areas, as these messages could be positively influential outside the campaign area. If these messages positively influenced persons in the control area, however, this potentially decreased our ability to detect differences between the campaign and control groups. Nevertheless, we found significant differences between these groups. Additionally, the metric of “potential reach” may overestimate the number of followers who actually viewed the content; however, this metric is an industry standard, given that is it often not possible to view actual reach when using influencers to deliver messaging (as was the case with this study). Finally, it is possible that those exposed to the campaign already had more positive attitudes toward the flu vaccine compared to the general population.

## Conclusions

This study shows that the approach of using influencers to deliver positive flu vaccine-related information is a promising strategy for communicating health information, changing flu vaccination perceptions, and possibly flu vaccine seeking behavior. Influencers can be an ideal tool for health communication if they already identify with a target audience, and their content uses the same language and style of speech that the audience uses. In this way, health campaigns can have a look, feel, and sound that will capture an audience’s attention. While the strategy of using unbranded native advertising techniques does not allow for an easy comparison of behavior change associated with campaign exposure, results showed encouraging results among those who reported awareness of a flu vaccine campaign on social media. We demonstrated that those who were exposed to the campaign were more likely to receive the flu vaccination and report positive flu vaccination perceptions. We feel that this presents an important step toward using innovative methodologies to communicate health information. This approach may be particularly important within the context of the COVID-19 pandemic. Health experts have expressed concern that the 2020–2021 flu season could present additional challenges to individual health, and could place strains on healthcare systems which may need to address both a COVID-19 pandemic and a flu season at the same time [47]. Given that African Americans and Hispanics are disproportionately affected by COVID-19 and are less likely to get the flu vaccine compared to other demographic groups, it is critical to employ new approaches that can deliver positive messages about vaccination to these groups [48].

Future studies should also examine how to address the challenge of evaluation using native advertising strategies which place the message at the center of the campaign, instead of a brand name. The marketing industry has acknowledged the fact that individuals are keenly aware when they are being advertised to; strategies to change behaviors must take into account new approaches to creating health campaigns [49–51]. Most studies using influencers to improve health behaviors have relied on campaign awareness or digital metrics to evaluate success. However, these metrics are often simply a reflection of digital ad spending, rather than an evaluation of behavior change [52, 53]. While the evaluation methodology employed in this study contained its limitations, it presents an alternative strategy of measuring success that merits further examination.

To effectively reach groups that show lower flu vaccination rates, we believe that national or large-scale flu campaigns must take a ground-up rather than top-down approach. By strategically leveraging community- and state-based influencers, and more tactically employing paid and earned media opportunities, flu campaigns can better reach priority audiences, increase positive perceptions about flu vaccination, and ultimately increase vaccination coverage.

## Supporting information

S1 File (PPTX)
