# Supplementary figures and images for: Using social media influencers to increase knowledge and positive attitudes toward the flu vaccine
Source: PLoS One. 2020 Oct 16;15(10):e0240828. doi: 10.1371/journal.pone.0240828 (PMC7567389; doi:10.1371/journal.pone.0240828)

## Slide 1
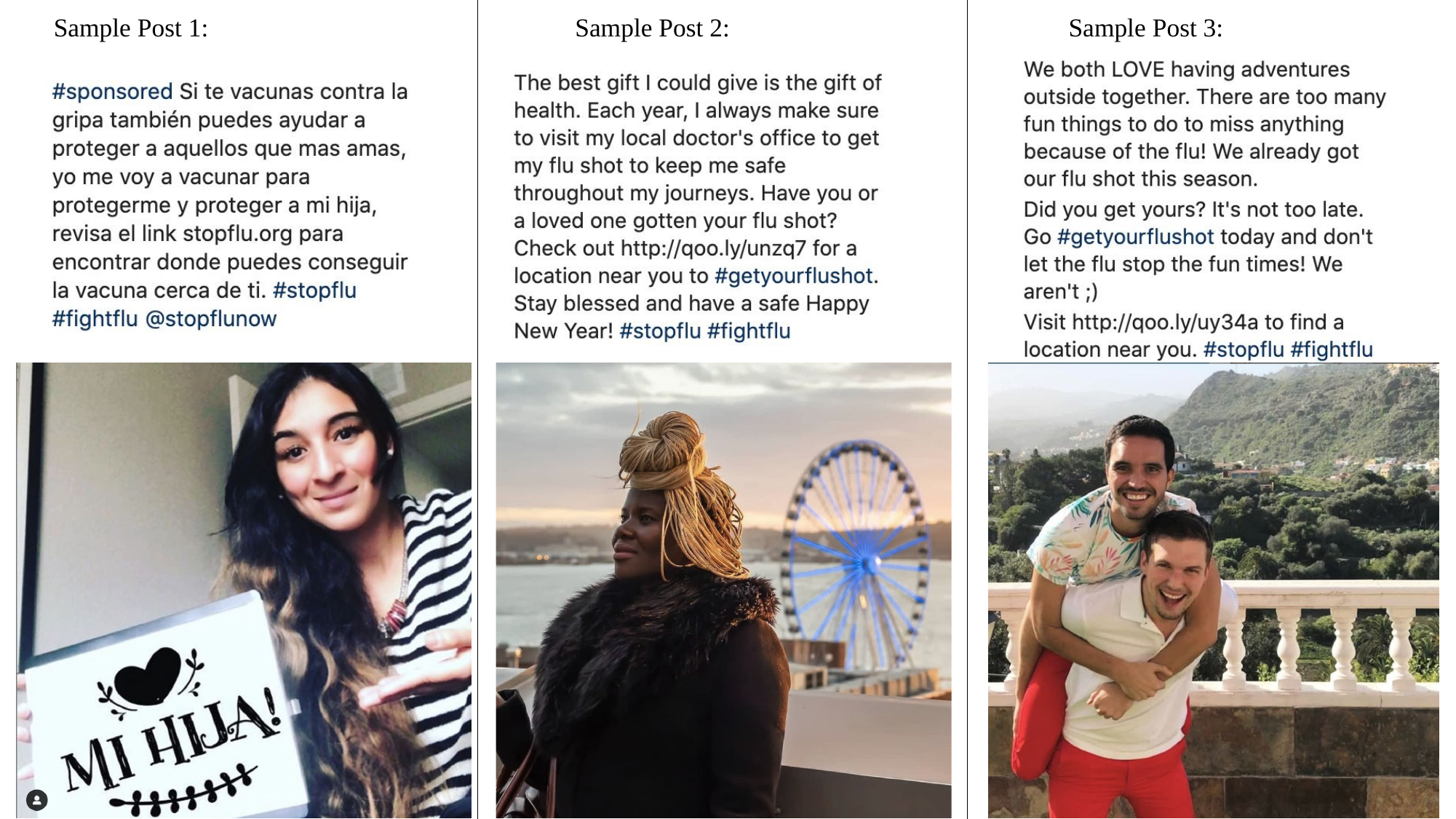

Sample Post 1:			 Sample Post 2: 			 Sample Post 3:

Supplement: S1 File — (PPTX) [file pone.0240828.s001.pptx]
